# The Midwife Problem in America

**Published:** 1911-04

**Authors:** 


### The Midwife Problem in America

AS the movement for the prevention of blindness from ophthalmia neonatorum progresses, it becomes more and more evident that the status of the American midwife must be raised. About half the births in the large cities of this country are attended by midwives and at present, with the exception of a few localities, these women engage in practice unregistered, unsupervised and uncontrolled.

All who have carefully studied the subject agree that for the present at least the midwife cannot be eliminated. But she may be trained and she should be put under state control. Great Britain has the same problem to deal with and the English plan recently adopted seems to work out well in practice.

The Central Midwives Board was established in England in 1902 by an act of parliament entitled the Midwives Act, "to secure the better training of midwives and to regulate their practice." The board does not itself undertake any of the training or instruction of midwives, this being carried out in maternity hospitals which are recognised by the board as training schools, or under the authority of a registered medical practitioner, accepted by the board as a teacher of midwifery.

The Midwives Act forbids any woman not holding a certificate issued by the Central Midwives board to practise as a midwife, and in order to be examined and licensed by this board, a candidate must present a diploma from a recognised training school, together with a certificate as to moral character. Having passed the Central Midwives Board examination and secured a license to practise the midwife is required to observe the rules and regulations adopted by the board. The rules adopted relate to her person, equipment, duties to mother and child, obligations with regard to disinfection, obligations in securing medical assistance, responsibility in returning notifications of births and deaths.

These rules are enforced through the agency of what are termed the local supervising authorities, which are councils of the counties or county boroughs throughout England and Wales. Violation of these rules is punishable by fine, suspension or even exclusion from practice.

Practising midwives are required to register annually with the local supervising authorities, their registrations being forwarded to the Central Midwives Board and the hope is entertained that in the near future they will also be required to take short post graduate courses of training at intervals of two or three years as long as they practise.

These are the provisions in England for the education, licensure and control of midwives. Study of the working of the

English system together with the results of its application suggest a possible solution of the midwife problem in Amercia in the form of a modification of the English method.

In this country the difficulties resolve themselves into the need of supplying facilities for the (1) education and training of midwives; (2) examination and licensure by the state; (3) supervision and control by local authorities.

Anything like adequate control of the situation makes all three of these provisions necessary; but most essential of all, since most fundamental, is the need of an educational provision for the midwife.

This might be secured if the State Board of Regents (in New York) or Boards of Education (in those states where regents do not exist) should establish an educational standard to which the training schools for midwives would have to conform whether existing at the time of the adoption of this provision or established subsequently. Obviously, these training schools would of necessity be integral parts of maternity hospitals. It could then be made illegal for any woman to practise midwifery who was not licensed by the state board of health, and this license would only be granted upon the presentation of a diploma from a recognised training school for midwives together with a certificate showing that she had passed the examinations of the state board of education and was of good moral character. We find an analogy to this in the present system of examining and registering nurses in many states in this country. In order to take a state examination, a nurse must present a diploma from a hospital training school which has been recognised by a state board of education as one which conforms to the stipulated requirements. The New York state education department employs a state inspector of nurses' training schools.

Educating, examining and registering the midwife is only half the battle for after these measures have been accomplished her practice must be regulated and she must be supervised and controlled. The state boards of health should adopt rules and regulations governing the practice of midwifery and should stipulate the details of the midwife's equipment.

The control of the midwife, however, and the enforcement of these rules would probably be most satisfactorily carried out by local health boards. The local boards of health should also be vested with power and authority to supervise the midwives themselves, their equipment and their work and to revoke licenses for violation of any of the regulations governing them or their practice.

It is evident that effective development of any such plan as is here suggested to improve the status of the American midwife can only be accomplished through the coöperation of state and local boards of health, state boards of education, physicians, those in charge of maternity hospitals or maternity wards of hospitals and last, but not least, the midwives themselves.

(Signed) C. C. V. B.

